# Attempts for Developing Novel Sugar-Based and Sugar-Free Sea Buckthorn Marmalades

**DOI:** 10.3390/molecules26113073

**Published:** 2021-05-21

**Authors:** Oana-Viorela Nistor, Carmen Alina Bolea, Doina-Georgeta Andronoiu, Mihaela Cotârleț, Nicoleta Stănciuc

**Affiliations:** Faculty of Food Science and Engineering, Dunărea de Jos University of Galati, 800008 Galati, Romania; Oana.Nistor@ugal.ro (O.-V.N.); Carmen.Bolea@ugal.ro (C.A.B.); Georgeta.Andronoiu@ugal.ro (D.-G.A.); Mihaela.Cotarlet@ugal.ro (M.C.)

**Keywords:** berry fruit, stevia, bioactive compounds, shelf-life, antioxidant activity

## Abstract

Sea buckthorn (*Hippophaė rhamnoides* L.) is recognized as a valuable source of vitamin C and antioxidants, frequently used as nutraceuticals and cosmeceuticals. In the present study, attempts are made to produce and characterize a novel type of marmalade using sea buckthorn berries processed at 102 °C into marmalade in two combinations, with whole cane or stevia sugar. Changes in the phytochemical profile, antioxidant activity, color, shelf-life, texture, microbiological, and sensorial characteristics were determined. The total carotenoids content in the marmalades were significantly different, with values of 0.91 ± 0.03 mg/g dry weight (DW) in the sample with whole sugar cane (C_z_) and 2.69 ± 0.14 mg/g DW in the sample with Stevia sugar (C_s_). Significant values of polyphenols were found, of 59.41 ± 1.13 mg GAE/g DW in C_z_ and 72.44 ± 2.31 mg GAE/g DW in C_s_, leading to an antioxidant activity of 45.12 ± 0.001 μMol Trolox/g DW and 118.07 ± 0.01 μMol Trolox/g DW, respectively. Accelerated storage study showed a decrease in all the phytochemicals, however no significant changes were found in antioxidant activity. Values of <100 CFU/g for yeasts and molds and <5 CFU/g for Enterobacteriaceae after 21 days of storage at the room temperature of the marmalades were determined. The sensorial and color results were more than acceptable. Overall, the results highlighted the potential of using sea buckthorn as a potential rich source of bioactive compounds to be used in the sugar-based products manufacturing.

## 1. Introduction

Sea buckthorn (*Hippophae rhamnoides* L.) is a yellow-to-orange berry fruit, which grows in low humid, alluvial gravel and wet landslips belonging to the Elaeagnaceae family. In Romania, sea buckthorn is widespread all over the fields, hills, and mountains [1]. Considering the high amounts of natural antioxidants including ascorbic acid, tocopherols, carotenoids, flavonoids, as well as health beneficial fatty acids, sea buckthorn is a prophylactic and a healing plant. Therefore, sea buckthorn is recognized to be used as alternative method in supporting the treatment or prevention of some illnesses such as: gastrointestinal disorders, obesity, diabetes, nervous system, and cardiovascular diseases [2].

The antioxidant activity of the specific bioactive compounds from sea buckthorn is involved in the improving of eyesight, hair, and skin quality, as well as it has antiproliferative effects for colon, liver, and breast cancer cells and leukemia cells [3]. In general, sea buckthorn berries are used as extracts, having many beneficial properties. For examples, besides antioxidative, anticancer, anti-inflammatory, anti-bacterial, and tissue protective [4]; recently, it has been demonstrated that the consumption of extract from sea-buckthorn berries leads to increase in number of selected types of stem cells (progenitor, endothelial, and lymphocytoid mesenchymal stem cells) in circulating blood [5]. However, the taste of sea buckthorn berries is not attractive for the consumers, being sour and astringent, due to the lack of natural sugars. Therefore, one of the proposed methods to improve the sensorial properties is to process the berries into jams and marmalade. Moreover, the short shelf life of fresh fruits and fruit off-season availability could be adversely affected if processing is not possible. Preserved food plays an important role modern consumers’ life. Marmalade is a very popular jellified product based on sugar or sweeteners with special nutritional qualities. It is well known that marmalade is a mixture, brought to a suitable gelled consistency, of water, sugars and fruit pulp, fruit purée, fruit juice, fruit peel or aqueous extract of fruit or any combination thereof, in every case obtained from citrus fruit, such that the quantity of citrus fruit used for every 1000 g of the finished product is not less than 200 g, of which not less than 75 g is obtained from the endocarp” [6]. The long stability of the marmalades could be achieved through a combination of thermal processing, control of water activity, and pH. The nutritional and biological properties of sea buckthorn need to be sustained by the addition of healthy sugar, such as whole cane or stevia sugar. Whole cane sugar does not involve the refining process and contains the natural molasses and the natural nutrients of sugar cane [7]. It mainly consists of fructose, glucose, and sucrose, and other minor nutrients which contribute to the biological and nutritional value such as: phenolics, proteins, minerals, and natural asparagines [8]. Stevia is an alternative sugar extracted from the leaves of *Stevia rebaudiana* plant. Considered as a non-nutritive natural sweetener, stevia represents a safe sugar substitute that does not affect human health [9], because it is non-metabolizable (non-caloric), non-fermentable, and does not cause dental caries and diabetes.

Therefore, the aim objective of this study was to contribute to the development of two types of sea buckthorn berries-based marmalades. Two variants of novel marmalades were obtained and characterized, based on phytochemicals, microbiological profile, and their overall acceptability. The results highlighted the potential of using sea buckthorn as a rich source of bioactives for jellified or sugar-based product development.

## 2. Results

### 2.1. Phytochemical Profile of the Samples

The phytochemical analyses were determined for the blank sample represented by the sea buckthorn pulp (C) and for the processed samples in both variants (sea buckthorn marmalade with whole cane sugar (coded as C_z_) and with stevia sugar (coded as C_s_), respectively). The results are shown in Table 1. The sea buckthorn pulp (C) showed a carotenoid content of 1.02 ± 0.052 mg/g dry weight (DW), with a lycopene of 1.09 ± 0.063 mg/g DW. Processing into marmalades lead to a reduction of carotenoids to 0.97 ± 0.03 mg/g DW in C_z_ and an increase in C_s_ to 2.69 ± 0.14 mg/g DW. The significant differences in carotenoids content in the two samples are due to the combined effect of sugars and thermal treatment. Therefore, it has been suggested that carotenoids need to be released from plant cell, solubilized into the lipid phase, and incorporated into mixed micelles with certain hydrolysates of lipids before absorption [10]. In addition to thermal treatment, an interaction between carotenoids and polysaccharides is needed to improve carotenoid bioaccesibility during digestion and absorption. Several studies reported that the network formed by de-esterified pectin molecules held together by hydrogen bonding and hydrophobic interactions induced by ultrasound treatment resulted in gel-like properties within the tomato pulp that inhibited lycopene digestion [11]. The results are highly corelated with the texture profile of sea buckthorn marmalades, with a denser and tighter gel network for C_z_, which caused a significant higher firmness value.

Therefore, it can be appreciated that the carotenoids content in terms of total carotenoids, β-carotene, and lycopene in C_z_ is lower due to the complex interaction of these bioactives with pectin and other macromolecular polysaccharides in the heat-treated samples, leading to significant differences in apparent viscosity of the samples. Additionally, it is well known that stevia is a sugar substitute with a considerable thermal (up to 80 °C) and pH (2–10) stability [12]. A protective effect of the steviol glycosides on carotenoids also could be suggested.

The total polyphenols content (TPC) showed values of 24.30 ± 0.37 mg GAE/g DW in C, 59.41 ± 1.13 mg GAE/g DW in C_z_ and 72.44 ± 2.31 mg GAE/g DW in C_s_. Mendelová et al. [13], as well as Ivanišová et al. [14], reported for different varieties of sea buckthorn juice values of TPC in the range of 13.03–25.35 g GAE/dm^−3^ DW. The values from the literature are lower compared to the presented one, which is expected, due to the benefits of the thermal treatment, which made the releasing of the bioactive compounds possible.

Total flavonoids (TFC) values were 31.14 ± 3.75 mg QE/g DW in C and 55.99 ± 3.79 mg QE/g DW in C_z_ and 67.35 ± 2.11 mg QE/g DW in C_s_, respectively.

The phytochemicals profile led to higher antioxidant activity in C of 121 ± 0.01 μMol Trolox/g DW, whereas the marmalades samples showed values of 45.12 ± 0.001 μMol Trolox/g DW in C_z_ and 118.07 ± 0.01 μMol Trolox/g DW in C_s_. The lower apparent viscosity of C_s_ sample led to a higher value for hydrophilic bioactives and antioxidant activity.

### 2.2. Phytochemicals Stability during Accelerated Storage

Both samples were packed into glass jars and stored to room temperature (21 ± 2 °C) for 21 days to evaluate the phytochemicals stability during storage. The large loss of nutrients and bioactive [15] ingredients induced by the storage of the C_z_ samples can affect the technological characteristics of the product, leading to the selection of appropriate processing parameters and optimization of the proper ingredients in the product.

The carotenoids decreasing (from 0.97 ± 0.04 to 0.35 ± 0.01 mg/g DW) after 21 days of storage for the samples with whole sugar addition could be generated by the acid formation, degradation of polysaccharides, or oxidation of reducing sugars. These results are in accordance with the findings of Kanwal, Randhawa, & Iqbal [16]. In C_s_, the heat-stability up to 200 °C, acid-stable, and non-fermentable characteristics of stevia sugar [17] induced the slightly decreased trend of carotenoids during storage. It could be seen that lycopene was severely affected by the storage. Similar to Li et al. [18] which determined a significant loss of lycopene in tomato hot pot sauce after 30 days of storage at 25 and 37 °C, for sea buckthorn marmalades, the storage decreased the lycopene content with 50% for the C_z_ samples and with almost 77% for C_s_. Specific water activity (0.86–0.95%) is an important factor which could influence the lycopene degradation alongside to temperature, light, oxygen. Therefore, the decrease in lycopene content could be explained by the reversion from *cis* to *trans* isomers which is generated by the unstable state of *cis* isomer [19].

For all samples, the initial total polyphenol content slightly decreased by the end of storage in the range of 13–17%. Thereafter, the downward trend continues by the end of storage for the TFC with significant values of 45% for C_z_ sample and 54% for C_s_. However, the TFC and the antioxidant activity values are higher than the results reported for various berries jams reported by [20]. The marmalades with Stevia sugar addition preserved better the bioactive compounds during storage, with a decrease in antioxidant values from 118 ± 0.01 to 106 ± 0.01 μMol Trolox/g DW.

### 2.3. Color Parameters

Table 2 shows the variation of color parameters. A slightly decrease in L* and b* values can be observed with increasing storage time for the C_s_ marmalade, probably due to carotenoids degradation. Similar findings were reported by Lele et al. [21] for sea buckthorn and quince juice. Significant decrease of almost 88% in lightness and yellow color can be noticed for the C_z_ sample, that could be assigned to the whole cane sugar addition, which is dark brown.

The addition of steviol glycosides lead the marmalade samples to more yellow (increase in the positive values of the b* parameter), and less red (positive value of the a* parameter), when compared to the samples with whole sugar cane. Similar findings were reported by [22] for low sugar apple preserves. It seems that stevia sugar addition and thermal treatment at 102 °C do not affect the color characteristics of the marmalade, sustained by the ΔE (11.41 ± 0.15), C* (33.81 ± 0.3) and h* (19.87 ± 0.6) values for the C_s_ sample, whereas a storage period of 21 days led to a slight decrease of these values. The significant decrease of chroma values of almost 80% correlated with the six-times higher increased values of hue are related to the whole sugar cane addition and its caramelization capacity during the thermal treatment for the C_z_ sample.

In summary, the color of sea buckthorn marmalade depends on the sugar and steviol glycosides content, as well as the thermal treatment impact. Addition of whole cane sugar induced darkness to the C_z_ marmalade samples, whereas samples using stevia sugar were brighter.

### 2.4. Texture Profile of Sea Buckthorn Marmalades

The results of the texture analysis are shown in Table 3.

Firmness is defined as the maximum force at the first compression. It is an expression of the gel resistance against deformation. In C_z_ sample, the added sugar reacted with pectin from sea buckthorn, leading to a denser and tighter gel network [23], which determined a 3.4-times higher firmness value, compared to the C_s_ sample. Similar firmness values were reported for shaped jelly marmalade with cranberry concentrate [24]. Sugar substitution determined lower values of adhesiveness, which is defined as the negative work between the two cycles. A similar behavior was observed by [25] for jam, in which a part of the sucrose was replaced with steviol glycoside. Cohesiveness is how well the product withstands a second deformation relative to its resistance under the first deformation [26]. The results show the highest cohesiveness value (0.6) for C_S_ sample. Springiness is measure of how well the sample recovers the deformation after the first compression cycle. For the sea buckthorn marmalade samples, slight differences can be noticed for this parameter. The instrumental texture analysis shows high correlation with sensorial analysis.

### 2.5. Microbial Evaluation of the Stored Marmalades

The microbiological analyses revealed that the sea buckthorn marmalades were microbiologically satisfactory during the storage period (Table 4 and Table 5).

The results were in accordance with Selvamuthukumaran & Khanum [27], who obtained a sea buckthorn marmalade rich in antioxidants, with non-detectable microbial population during 8 months of storage at room temperature.

### 2.6. Sensorial Evaluation of the Marmalade Samples

**Figure 1:**
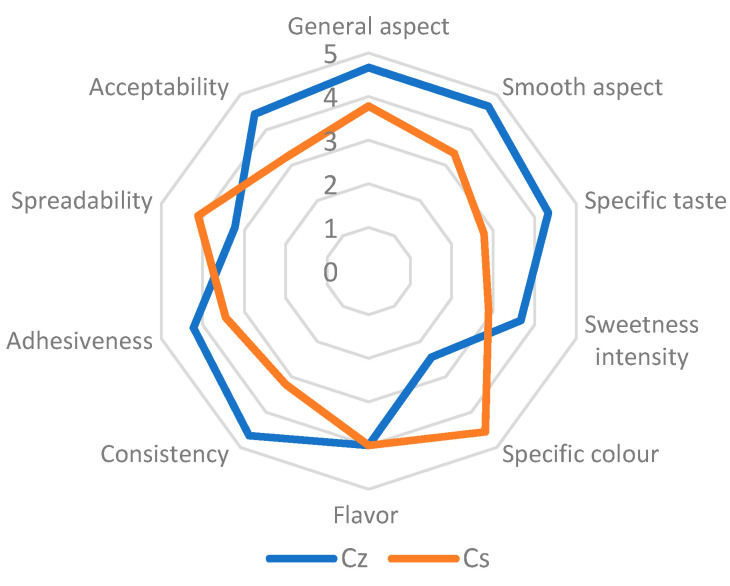
Sensorial analysis of sea buckthorn marmalades.

The general aspect of the marmalade with whole cane sugar (C_z_) gained the highest value of 4.67, while the marmalade with stevia sugar (C_s_) registered a 3.77 score. The most preferred sample was C_z_, which registered for all the attributes the highest scores excepting the color, as it can be observed from the Figure 1.

The C_s_ marmalade color was more appreciated with 4.56 points than the C_z_ sample with 2.44 points. This fact could be explained by the whole sugar cane directly influence on the C_z_ sample color. The panelists appreciated the taste and the textural characteristics represented by the consistency and adhesiveness of the marmalade with whole sugar cane. Similar results were obtained by Emelike & Akusu [28] for some marmalades and jams from tropical fruits and by Selvamuthukumaran & Khanum [27] for sea buckthorn jam. There were significant differences between the marmalade types of color, texture, spreadability, and taste. It is a notable the fact that stevia sugar has influenced the specific taste of sea buckthorn marmalade. No significant differences (*p* > 0.05) were determined between the sensorial attributes of the marmalade samples.

## 3. Materials and Methods

### 3.1. Chemicals

Ethanol, Folin–Ciocalteu’s reagent, sodium carbonate, ABTS (2,2-azino-bis(3-ethylbenzothiazoline-6-sulfonic acid) diammonium salt), quercetin, aluminum chloride, potassium chloride, sodium acetate, and petroleum ether were purchased from Sigma Aldrich (MilliporeSigma, Steinheim, Germany).

### 3.2. Plant Material

Freeze ecological sea buckthorn were purchased from a local supermarket from Galati, Romania in November 2020. The sea buckthorn was thawed to the room temperature, then sorted and washed under cold water jet.

### 3.3. Marmalade Making

Two types of marmalades were obtained based on 1000 g of sea buckthorn berries and 500 g of whole cane sugar (SanoVita, Valcea, Romania) or the correspondent of the sweetening power of 62.5 g of stevia sugar (Nutrivita, Ilfov, Romania) as well as 0.5% apple pectin (Pektin, Nature Cookta, Budapest, Hungary). The fruits were crushed in a blender for 3 min at 1000 rpm with a Philips HR2100/40 blender, EC (European Community). Furthermore, the obtained puree was blended with apple pectin, whole cane sugar, or stevia sugar for 7 min at the same speed. In between each addition, the substance was stirred continuously. The mixes were heated in a non-stick electric pot (Multicooker Philips HD3037/70, Hamburg, Germany) at 102 °C for one hour in the sugar case and half an hour for stevia. The final consistency of the marmalade was tested by the cold test, using a cold plate to determine the extent of the gelation. The marmalade was packed into glass jars and stored at room temperature (21 ± 2 °C).

### 3.4. Phytochemical Profile

#### 3.4.1. Extraction of Bioactive Compounds

For total polyphenolic and flavonoidic content, the sea buckthorn marmalades (1 g) were homogenized with 8 mL ethanol (70%) and stirred using an orbital shaker at room temperature for 24 h to extract the biological active compounds. The obtained extract was centrifuged at a speed of 5000× *g* for 30 min. For the phytochemical profile were used three samples for each marmalade.

#### 3.4.2. Determination of Total Phenolic and Total Flavonoid Content

The total polyphenolic content (TPC) and flavonoids content (TFC) were determined as described by [29].

#### 3.4.3. Antioxidant Activity by ABTS Assay

The ABTS+ radical’s method was employed as described by Xu, Chen, & Guo [30]. In brief, ABTS radical cation (ABTS∙+) was produced by reacting equal volumes of 7 mM ABTS stock solution with 2.45 mM K_2_S_2_O_8_ and allowing the mixture to stand in dark for 16 h before use. Further, aliquots of 1 mL ABTS∙+ solution was diluted with 35 mL methanol to get absorbance of 1.12 ± 0.02 at 734 nm. A volume of 2.85 mL of the ABTS∙+ solution and 0.15 mL of the sample could react for 2 h in a dark room before its absorbance at 734 nm was measured. The ABTS∙+ antioxidant activity of the samples was expressed as mM Trolox equivalents/g DW based on the calibration curve.

#### 3.4.4. Extraction of Carotenoids from Sea Buckthorn Marmalades

An amount of 2 g of sea buckthorn marmalade samples was homogenized with 10 mL of petroleum ether. Then, the extraction was further performed by ultrasonication for 30 min (MRC Scientific Instruments, Essex, United Kingdom). The ultrasonic bath is equipped with a digital control system of sonication time, temperature, and frequency. The extraction was performed at a constant frequency of 40 kHz and power of 100 W. The resulting supernatant was collected and centrifuged at 9000× *g*, at 10 °C for 10 min.

#### 3.4.5. Carotenoids Content

Carotenoids content, in terms of total carotenoids, β-carotene and lycopene were determined by spectrophotometric method. The absorbance was measured at 470 nm, 450 nm, and 503 nm. The amount of carotenoids was calculated according to the equation [31]

Carotenoids (mg/g) = A·M_W_·D_f_/(M_a_·L) (1)

where A—absorbance of the petroleum ether phase at corresponding wavelength; Mw—molecular weight, D_f_—sample dilution rate, M_a_—molar absorptivity (2500 L mol^−1^ cm^−1^, 2590 L mol^−1^ cm^−1^ and 3450 L mol^−1^ cm^−1^, respectively), and L—cell diameter of the spectrophotometer (1 cm).

### 3.5. Color

The color of the marmalades samples was measured using a MINOLTA Chroma Meter CR-410 (Konica Minolta, Osaka, Japan) fitted with a granular accessory, after standardization with a white calibration plate according to the equipment specifications. The determined parameters were L* (lightness/darkness), a* (red/green) and b* (yellow/blue). The total color difference (Δ*E*) between samples was calculated according to Equation (2)

(2) $$\Delta E=\sqrt{{\left(L_{0}^{*}-{\;L}^{*}\right)}^{2}+{\left(a_{0}^{*}-a^{*}\right)}^{2}+{\left(b_{0}^{*}-b^{*}\right)}^{2}}$$

Subscript 0 refers to the color of the fresh sample. The hue angle (h*), visual color appearance, and chroma (C*), color intensity, were calculated according to Equations (3) and (4), using the formula

(3) $$C^{*}=\sqrt{a^{*2}+b^{*2}}$$

(4) $$h^{*}={\;\tan}^{-1}\left(\frac{b^{*}}{a^{*}}\right)$$

Three replicates were carried out for each sample.

### 3.6. Texture

Texture Profile Analysis (TPA) method, achieved with a CT3-1000 Texture Analyzer (Brookfield Ametek, Middleborough, MA, USA) was used to evaluate the textural properties of the samples. The hot samples were poured into cylindrical plastic containers, with 43 mm diameter and 40 mm high (the sample height was approximatively 30 mm). After reaching the room temperature, the samples were introduced into a refrigerator and kept until the next day. Before testing, the samples were equilibrated at room temperature and subjected to a double penetration test (texture profile analysis—TPA), until the target distance of 10 mm was reached. For testing, a cylindrical acrylic probe, with 12.7 mm was used. Test speed was 1 mm/s, trigger load was 0.067 N and the lode cell was 1000 g. TexturePro CT V1.5 software was used to register the force-deformation parameters and to calculate the textural parameters: firmness, adhesiveness, cohesiveness, and springiness. Four determinations for each sample were done.

### 3.7. Microbial Shelf-Life Examination of Sea Buckthorn Marmalades

To count the Enterobacteriaceae, yeasts and molds, the standards ISO 21528-2:2017 [32] and ISO 21527-2:2008 [33] were used.

### 3.8. Sensorial Evaluation

The sensorial evaluation of the marmalades was conducted as described by Emelike & Akusu [28] using 10 panelists, aged 27–44 years, selected from the university community, whose experience in sensory evaluation in this type of product were acknowledged.

The panelists were trained before the final sensorial evaluation. Training happened over a period of one months with one session per week. Each panelist tested a number of no more than four samples for test with breaks included between tests. The aim of the training was to be familiarized with the marmalade attributes and to learn the specific terminology to describe the samples. The samples were stored at the room temperature and tested after a week from the production point, packaged in transparent packages and presented in a coded manner. The sensorial attributes of the samples were: general aspect, smooth aspect, specific taste, sweetness intensity, specific color, flavor, consistency, adhesiveness, spreadability, and acceptability. All the terms were defined to the panelists. The panelists were requested to observe and taste each sample as coded and grade them based on a five-point hedonic scale, where 1 represents the lowest grade and 5 the highest grade. After evaluating each sample, they rinse their mouth with potable water to hesitate the taste interference.

### 3.9. Statistical Analysis

The results referring to phytochemicals, texture, color, and sensorial parameters were statistically analyzed using a Minitab 19 software–Free Trial (Ottawa, ON, Canada) to identify significant differences. The Tukey’s test with a 95% confidence interval was applied.

## 4. Conclusions

Attempts were made in this study to identify proper technological approaches for novel sea buckthorn sugar-based and sugar-free marmalades, based on phytochemicals, textural, color, sensorial, and microbiological evaluation of the final products. Two different types of sugars were used—whole cane sugar and stevia sugar, respectively—based on developing food for the consumption of low calories foods. Significantly different phytochemicals and color profiles were highlighted for the marmalades, with a higher content of carotenoids and polyphenols, and consequently a higher antioxidant activity in marmalade with Stevia sugar addition, due to the specific network formed. Lower phytochemical content was found in marmalade with whole cane sugar due to the complex interaction of bioactives with pectin and other macromolecular polysaccharides in the heat-treated samples, leading to significant differences in apparent viscosity. Both types of marmalades could be safely stored up to a period of 21 days at room temperature, with no significant decrease in color parameters. However, phytochemicals were found to decrease during accelerated storage test, with a highest value for lycopene. The textural profile analysis suggested that the sugar substitution with Stevia determined lower values of the firmness and adhesiveness parameters. Sensorial analysis showed significant differences between the taste and the color of the samples influenced by both types of added sugar.

## Figures and Tables

**Table 1:** Phytochemical properties of the marmalade samples.

Code	β-Caroten, mg/g DW	Lycopene, mg/g DW	TFC, mg EC/g DW	TPC, mg EAG/g DW	ABTS, μMol Trolox/g DW
C	1.02 ± 0.05 ^a^	1.09 ± 0.06 ^a^	31.14 ± 3.75 ^a^	24.30 ± 0.37 ^a^	121.12 ± 0.01 ^a^
C_z0_	0.91 ± 0.03 ^a^	0.13 ± 0.02 ^a^	55.99 ± 3.79 ^a^	59.41 ± 1.13 ^a^	45.12 ± 0.001 ^a^
C_z7_	0.52 ± 0.005 ^a^	0.12 ± 0.002 ^a^	51.25 ± 0.79 ^a^	57.07 ± 2.99 ^a^	46.34 ± 0.01 ^a^
C_z14_	0.43 ± 0.002 ^a^	0.09 ± 0.004 ^a^	33.98 ± 1.94 ^a^	54.34 ± 3.13 ^a^	43.03 ± 0.01 ^a^
C_z21_	0.35 ± 0.01 ^a^	0.07 ± 0.001 ^a^	30.77 ± 0.09 ^a^	51.67 ± 2.90 ^a^	42.08 ± 0.02 ^a^
C_s0_	2.69 ± 0.14 ^a^	1.20 ± 0.013 ^a^	67.35 ± 2.11 ^a^	72.44 ± 2.31 ^a^	118.07 ± 0.01 ^a^
C_s7_	2.28 ± 0.04 ^a^	0.54 ± 0.07 ^a^	63.60 ± 1.67 ^a^	66.55 ± 0.77 ^a^	114.04 ± 0.03 ^a^
C_s14_	2.13 ± 0.05 ^a^	0.31 ± 0.015 ^a^	41.70 ± 1.94 ^a^	61.14 ± 0.78 ^a^	109.10 ± 0.01 ^a^
C_s21_	2.03 ± 0.01 ^a^	0.27 ± 0.001 ^a^	31.02 ± 1.67 ^a^	59.64 ± 3.09 ^a^	106.02 ± 0.01 ^a^

TPC, total phenolic content; TFC, total flavonoid content; C—sea buckthorn blank samples; C_z_—sea buckthorn marmalade with whole cane sugar samples; C_s_—sea buckthorn marmalade with stevia sugar samples, 0, 7, 14, 21—the days of the analysis. Values are represented as mean ± standard errors. The same superscript letter (^a^) means no significant differences (*p* = 0.05) between the parameters for the same column.

**Table 2:** Color parameters of marmalades.

Code	L*	a*	b*	ΔE	C*	h*
C	50.35 ± 0.05 ^a^	12.94 ± 0.1 ^a^	36.41 ± 0.5 ^a^	-	38.63 ± 0.3 ^a^	20.33 ± 0.2 ^a^
C_z0_	20.5 ± 0.15 ^a^	7.27 ± 0.01 ^a^	3.7 ± 0.05 ^a^	44.65 ± 0.3 ^a^	8.15 ± 0.1 ^a^	112.78 ± 0.8 ^a^
C_z7_	20.44 ± 0.25 ^a^	7.13 ± 0.03 ^a^	3.64 ± 0.1 ^a^	44.74 ± 0.5 ^a^	8.00 ± 0.15 ^a^	112.34 ± 0.9 ^a^
C_z14_	20.34 ± 0.1 ^a^	7.41 ± 0.05 ^a^	4.62 ± 0.05 ^a^	44.07 ± 0.3 ^a^	8.72 ± 0.2 ^a^	92.4 ± 0.6 ^a^
C_z21_	20.27 ± 0.15 ^a^	7.22 ± 0.1 ^a^	4.21 ± 0.1 ^a^	44.44 ± 0.4 ^a^	8.35 ± 0.05 ^a^	98.37 ± 0.4 ^a^
C_s0_	40.01 ± 0.15 ^a^	11.09 ± 0.03 ^a^	31.95 ± 0.2 ^a^	11.41 ± 0.15 ^a^	33.81 ± 0.3 ^a^	19.87 ± 0.6 ^a^
C_s7_	40.13 ± 0.05 ^a^	11.01 ± 0.2 ^a^	31.06 ± 0.1 ^a^	11.7 ± 0.1 ^a^	32.95 ± 0.2 ^a^	20.3 ± 0.8 ^a^
C_s14_	41.31 ± 0.03 ^a^	11.17 ± 0.3 ^a^	34.32 ± 0.15 ^a^	9.47 ± 0.2 ^a^	36.09 ± 0.5 ^a^	18.63 ± 0.2 ^a^
C_s21_	41.35 ± 0.2 ^a^	11.16 ± 0.2 ^a^	36.35 ± 0.2 ^a^	9.34 ± 0.15 ^a^	36.4 ± 0.5 ^a^	17.42 ± 0.1 ^a^

L* (lightness/darkness), a* (red/green) and b* (yellow/blue), ΔE—total color difference, h*—hue angle, C*—chroma, color intensity. Values are represented as mean ± standard errors. Means in a row bearing the same superscript letters do not differ significantly.

**Table 3:** Texture parameters values for marmalade samples.

Texture Parameter	C_s_	C_z_
Firmness, N	1.25 ± 0.03 ^b^	4.31 ± 0.76 ^b^
Adhesiveness, mJ	1.98 ± 0.2 ^b^	3.56 ± 0.96 ^b^
Cohesiveness	0.6 ± 0.05 ^b^	0.28 ± 0.02 ^b^
Springiness, mm	8.1 ± 0.82 ^a^	8.84 ± 0.68 ^a^

The values are presented as means ± standard deviation. Different superscript letters (a and b) mean a significant difference at (*p* < 0.05) among different parameters for the same column.

**Table 4:** Yeasts and molds enumeration during storage, CFU/g.

Sample	Storage Period, Days			
	0	7	14	21
C_s_	<100	<100	<100	<100
C_z_	<100	<100	<100	<100

**Table 5:** Enterobacteriaceae enumeration during storage, CFU/g.

Sample	Storage Period, Days			
	0	7	14	21
C_s_	<5	<5	<5	<5
C_z_	<5	<5	<5	<5

## Sample Availability

Samples of the compounds are available from the authors.
